# Prioritization and selection of high fuelwood producing plant species at Boset District, Central Ethiopia: an ethnobotanical approach

**DOI:** 10.1186/s13002-021-00474-9

**Published:** 2021-08-16

**Authors:** Tinsae Bahru, Berhane Kidane, Amsalu Tolessa

**Affiliations:** 1 Central Ethiopia Environment and Forest Research Center (CEE-FRC), Addis Ababa, Ethiopia; 2 Forest Products Innovation Research and Training Center (FPIRTC), Addis Ababa, Ethiopia

**Keywords:** *Acacia* species, Boset, Ethnobotany, Fuelwood, Management

## Abstract

**Background:**

In Ethiopia, about 92.3% of all the fuelwood (firewood and/or charcoal) is consumed for cooking, heating and lighting purposes by domestic households and the demand is growing from 10 to 14%. However, there are little/no practical experiences or documented indigenous knowledge on how traditional people identify and select high fuelwood producing plant species with short rotation periods at Boset District. Therefore, the present study was aimed at: (1) selecting and documenting high fuelwood producing plant species at Boset District; (2) identifying major predictor variables that influence the prioritization and selection of species; and (3) develop a Generalized Linear Model (GLM) to predict the selection of species.

**Methods:**

A total of 96 informants comprising 59 men and 37 women between the ages of 18 and 81 were sampled. Data were collected using structured interviews, guided field walk, discussions and field observations.

**Results:**

Collected data indicated that 88.5% of the informants involved in firewood collection, while 90% practiced charcoal making. A total of 1533.60 Birr per household on average was earned annually from this activity. A total of 25 fuelwood producing plant species were identified and documented at Boset District. Of these, *Acacia senegal, Acacia tortilis* and *Acacia robusta* were the three best prioritized and selected indigenous high fuelwood producing species. *Prosopis juliflora*, *Parthenium hysterophorus*, *Azadirachta indica*, *Calotropis procera, Cryptostegia grandiflora, Lantana camara* and *Senna occidentalis* further grouped under introduced fuelwood species. Prediction of GLM assured sampled *Kebeles* and source of income generated from fuelwood species positively and significantly (*p* < 0.001) related to selection of species. Higher efficiency to provide energy and heat; little or no smoke or soot; easier to cut and split the wood and easier availability were some of the main selection criteria.

**Conclusions:**

This study provides valuable information in selecting and documenting of high fuelwood producing plant species for proper management and sustainable use at Boset District. The three most selected species (*A. senegal, A. tortilis* and *A. robusta*) should be further evaluated at laboratory to determine their calorific value and combustion characteristics.

## Introduction

Fuelwood (firewood and/or charcoal) is the major source of energy both for domestic supply and commercial uses in east Africa. Hence, it accounts for about 85% of the total energy consumed and for over 95% of the total wood products used in east Africa [1]. Out of this energy supply, the main source of fuelwood is forest resources, which accounted for over 99% of the fuelwood consumed [1]. Consequently, fuelwood is the main source of cooking, heating and lighting in the region. Almost 100% of the rural population as well as 80% of the urban population rely upon fuelwood as their main source of domestic energy [1]. In line with this, a higher demand of energy in Ethiopia is growing from 10 to 14% [2]. However, according to the UNEPs study such energy source is mainly rely on fuelwood sources, which includes firewood and charcoal as well as agricultural crop residues and animal dung. As a result, a high demand for solid wood (firewood and charcoal) accompanied by population pressure causes reduced access to fuelwood species. Furthermore, overexploitation of these fuelwood species is associated with deforestation, land degradation and climate change. Following this event, the demand for charcoal consumption in the urban areas and firewood and agricultural residues in the rural setting becomes very high and dramatically rising [2].

Similarly, according to WBISPP [3] report about 92.3% of all the fuelwood is consumed in Ethiopia for cooking and lighting purposes by domestic households. Consequently, the most important sources of fuel, which are the necessities for human kind, are fuelwood (charcoal and firewood), petroleum and peat. Of these, wood makes an outstanding fuel as it is 99% flammable if completely dry [4, 5]. It is the cheapest, the most suitable and accessible energy source in many rural areas [6, 7]. For example, Abbiw [6] reported that 90% of the harvested wood is used for fuelwood. However, an inefficient and wasteful method of traditional open fire cooking and the low efficiency cooking stoves accounts mainly for the consumption of relatively a higher proportion of fuelwood. At the same time, such traditional stoves causes a lot of smoke and soot which is associated with health problems. Women and children also spend a lot of time and energy to collect fuelwood from long distance. So, to combat the problem of deforestation, designing efficient and energy-saving improved stoves is one of the practical solutions in many developing countries. Although the majority of wild wood-based plant species can be used as a source of fuel for indigenous peoples, many species are recognized for particular burning qualities [7].

Charcoal is a domestic fuel for cooking and heating [4, 8–11]. Due to this reason, it is a common source of fuelwood in urban centers. In the absence of fossil fuel, charcoal is more advantageous and much preferred fuelwood than firewood due to being of lighter weight, less bulky and more compact, thereby easier to store indefinitely and cheaper to transport [6, 11]. It is more efficient and produces a steady heat with little or no smoke or soot [4–8]. On the contrary, a lot of processes are required to make charcoal locally using simple traditional mound kilns. During charcoal preparation, about 50% of the wood’s energy is wastefully burned away during carbonization process [6, 9, 11]. With this, one ton of charcoal is produced from about 5 tons of fuelwood [10]. Due to this reason, firewood is better than charcoal if the transport distance is short. The long distance transportation further makes charcoal more expensive compared to firewood. Consequently, extensive woodland has to be cleared to meet the high charcoal demand. High valued fuelwood species for charcoal production such as *Acacia* and other indigenous species are severely degraded and even endangered. Moreover, charcoal making causes many accidental fires on forests and thereby devastates the forest resources associated with a huge loss of biodiversity. At the same time, both charcoal harvesting and accidental and/or deliberate forest fire further contribute to deforestation, land degradation and climate change [12].

As a result, there are a number of limitations are found in optimum production and sustainable use of fuelwood in Ethiopia. Problems of fuelwood collection, transportation and storage as well as availability are some of the main factors. In addition, the ever-increasing demand for optimum and sustainable production of fuelwood coupled with efforts to mitigate greenhouse gas emissions and thereby adapt climate change as well as forest degradation especially for indigenous species has needs very urgent call and attention for the identification, selection as well as large scale development and promotion of forest plantation. Hence, in order to solve such problems producing sufficient and sustainable quantities and expansion of fuelwood from fast growing fuelwood species with short-term rotation periods are an option for balancing the demand and supply of fuelwood. To achieve this objective, superior fuelwood species characterized by higher survival rate, better growth performance and disease resistance should be promoted for short-rotation fuelwood production [13]. This is because fuelwood will continue to be the most reliable source of energy in rural communities in Africa so that there is a need to expand its supply so as to satisfy the demand. As a result, a lot of effort has been done over the last a few decades to establish plantations particularly short-rotation fuelwood species such as *Eucalyptus* species. This, in turn, helps to solve the ever-increasing imbalance of demand and supply of fuelwood species associated with population pressure and expansion of urbanization. Thus, these short-rotation fuelwood species not only address the shortage of fuelwood demand for the long-term problem of the society but also they play a significant role in mitigating greenhouse gas emissions and thereby adapt climate change as well as forest degradation especially for indigenous species. Due to these and other related reasons fast growing fuelwood species having short-rotation periods currently rapidly expanding in various parts of Ethiopia in order to solve the fuelwood demand both in rural and urban areas. Consequently, many small-scale farmers in Amhara region widely plant and expand the *Eucalyptus* species (e.g. *E. globulus* and *E. camaldulensis*) and *Acacia decurrens* in their farmlands [14] by converting from cultivation of crops.

However, there is a knowledge gap on selecting and prioritizing the most preferred suitable fuelwood species to expand the plantation forests both in small-scale and large-scale plantations through the application of arboriculture and silviculture. Furthermore, selected species should be further evaluated under laboratory to check their calorific value and combustion characteristics. Of course, there are previous works that reports the calorific value and combustion characteristics of prioritized species (e.g. *A. senegal* [15] and *A. tortilis* [16]). But, the calorific value and combustion characteristics for a particular species might be varied due to various environmental factors such as geographical location, soil types, altitude, rainfall, etc. Therefore, the present study was aimed at: (1) selecting and documenting high fuelwood producing plant species with short-rotation period to satisfy the high demand of fuelwood at Boset District; (2) identifying major predictor variables that influence the identification, prioritization and selection of fuelwood species; (3) develop a Generalized Linear Model (GLM) to predict or explain the selection and documentation of the most preferred fuelwood species in the study area; and (4) suggesting the best three prioritized and selected fuelwood species for further evaluation of their calorific value and combustion characteristics at laboratory.

## Materials and methods

### Study area

**Fig. 1:**
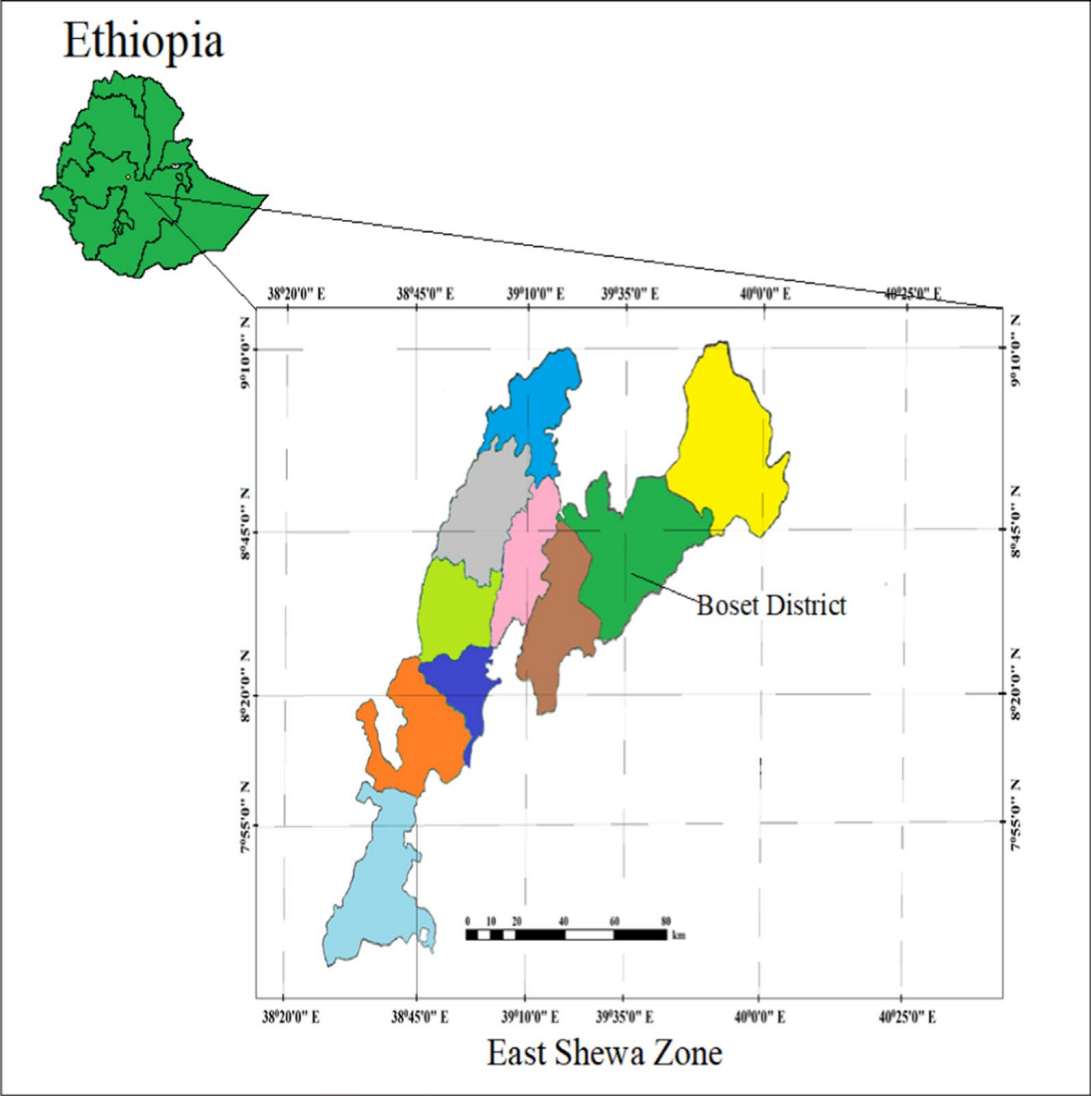
Location of Boset District at East Shewa Zone of Oromia National Regional State, central Ethiopia. The Zonal map was modified and adopted from Hundera et al. [17]

An ethnobotanical study was conducted in Boset District, East Shewa Zone of Oromia National Regional State, central Ethiopia (Fig. 1). It is located in the Great East African Rift Valley. The capital town of Boset District, *i.e*., Olenchiti town is 25 km far from Adama town, while 99 km far from Addis Ababa, in the eastern Ethiopia. Olenchiti town is located between latitudes 37P 0547246 and longitudes UTM 0957166 within an altitude of 1465 m a.s.l.

The three study sites (*Kebeles*) are found around the Olenchiti town. The term *Kebele* is part of a District and the smallest administrative division or structure in Ethiopia. The three selected study *Kebeles* from Boset District (Borchota, Geri and Tedecha) are located around the Olenchiti town. These *Kebeles* are 12, 11 and five km far from Olenchiti town in their respective orders. Based on the 2007 Census conducted by the CSA, Boset District has a total population of 141,196 out of whom 73,443 are men and 67,753 are women [18]. Of these, a total of 114,687 populations live in the rural setting that having 60,107 male and 54,580 women. The study area is generally practice mixed agriculture that comprising both livestock raising and crop production.

Boset District, which is located within the Great East African Rifit Valley, is characterized by semi-arid climate or *Qolla Zone.* Its mean annual rainfall is ranged from 600 to 900 mm, while the mean annual temperature is 26–34 °C [19]. This document further reported that the dominant soil types of the study area include brown (*Dalecha*) soil 30%, 13% black soil, 25% sand, 25% clay, 5% volcanic soil and the rest 2% other soil types. The main land use types are 28.7% agricultural land, 7% grazing land, 0.1% forest land, 51.9% uncultivated land and 1.47% construction and other uses [19]. The area is located within the Inter-Tropical Convergence Zone, which makes both temporal and spatial variability in rainfall, humidity and temperature [20]. Rainfall is bimodal with two distinct seasons, i.e., the *short* rains (*Belig* rain) between February and April and the *long* rains (*Meher* rain) between July and September [20]. The vegetation of the study area lies under the Somalia-Maasi center of endemism [21]. Sebsebe and Friis [22] further described that out of the nine vegetation types of Ethiopia, the vegetation type of the study area is classified under *Acacia-Commiphora* woodland. Accordingly, this vegetation type is characterized by drought-resistant trees and shrubs, either deciduous or with small, evergreen leaves. The trees and shrubs in this vegetation type include species of *Acacia*, *Commiphora, Balanites, Capparis, Combretum* and *Terminalia.*

### Methodology

A two-stage sampling design was followed for conducting the in-depth ethnobotanical study at the study site. In the first stage, a potential District, i.e., Boset District was purposively selected out of ten Districts from east Shewa Zone, Oromia National Regional State, central Ethiopia. The basis of selection mainly relied upon harvesting intensity of firewood collection and/or charcoal production in the area by firewood collectors and/or charcoal makers. Accordingly, the selection was conducted based on previous information/data by the east Shewa Zone Environment, Forest and Climate Change Authority. Following this, three potential *Kebeles* from Boset District were purposively selected out of 14 *Kebeles*. During the selection process, Boset District Agricultural and Natural Resources Office Head, District Natural Resources Expert, *Kebele* Development Agents (DA) and *Kebele* Officials were actively involved and gave their own suggestion. Three selected *Kebeles* were Borchota, Geri and Tedecha. These *Kebeles* were known with severe harvesting of firewood and/or charcoal production. In the second stage, a reconnaissance survey and pre-test at three selected study sites was conducted in order to obtain an impression about commonly used high fuelwood producing plant species by charcoal makers, fuelwood collectors, pastoralists, farmers, religious and community leaders as well as local communities to identify and select informants [23]. Moreover, identified charcoal makers and fuelwood collectors were invited to suggest other charcoal makers and fuelwood collectors. This is because these informants had better experience, knowledge and skills associated with their day-to-day activities [23]. Informants’ selection was mainly based on their local knowledge, skills and daily practices on firewood collection and charcoal production, which was evaluated during pre-test survey. In this insight, informants within different age, gender, occupation, wealth status and social groups were included from different households and considered as household heads in this particular ethnobotanical study. This is because indigenous knowledge considerably varies among age, gender, occupation, wealth status and different social groups [7, 23–25]. At the same time, *Kebele* Officials and Development Agents (DA) were actively involved and participated during the selection process as they have a better experience and socio-economic information of the informants.

Accordingly, 32 informants and key informants from each sampled *Kebele* were selected. Key informants were informants, who had better knowledge, skills and practices of the local community on harvesting of firewood and charcoal production. Informants and key informants across these *Kebeles* were listed and selected based on previous practices, knowledge and experiences on firewood collection and charcoal making. Following this, a total of 96 informants (59 men and 37 women) from three sampled *Kebeles* of Boset District were selected and each informant was interviewed with structured questionnaire for the in-depth ethnobotanical study (Table 1). As much as possible, all the households available or present in the *Kebele* at the time of this study were interviewed. However, more females were not included as expected since they were not available or because of other unknown socio-economic or cultural reasons. Following the methods by Cotton [7], Bahru et al. [23], Cardoso et al. [26], Cunningham [27] and Martin [28] structured interview, guided field walk, individual and group discussions as well as direct researcher’s observation were applied to identify high fuelwood producing plant species. During data collection, basic indigenous knowledge on firewood collection and charcoal making including their socio-economic data (population demography); informants’ livelihoods from agricultural products, livestock husbandry, forest and forest products, income from firewood collection and charcoal making as well as other income generating activities. Other important information included mainly sources of energy for cooking, heating and lighting; potential tree/shrub species with high fuelwood production and their major features; marketing system of fuelwood sources; good opportunities and major challenges to properly manage and sustainably utilize high fuelwood producing species and other related aspects were further investigated in detail. During the guided field walk, specimens of fuelwood producing species reported and identified by informants were identified at field and cross-checked at the National Herbarium, Addis Ababa University, Ethiopia. Then, identified and listed species from each *Kebele* were further ranked from 1 to 5 using simple preference ranking following Cotton [7] and Martin [28]. Following this, 10–15 key informants were selected and the paired comparison method was applied. Thereafter, the best three selected species from each *Kebele* after preference ranking were further prioritized again using paired comparison method following Cotton [7] and Martin [28]. Finally, the first ranked potential species for firewood collection and charcoal making from each *Kebele*, *i.e*., three species from three *Kebeles* were prioritized and recommended for laboratory analysis to be evaluated their potential for fuelwood values. As a whole, this ethnobotanical field method was effectively applied following Bahru et al. [23, 24], which are conducted for other related ethnobotanical studies with diverse species in and around the semi-arid Awash National Park. Eventually, collected ethnobotanical data were feed to computer excel sheet and summarized, quantified and presented using non-descriptive statistical methods such as graphs, tables and percentages. All the statistical data analysis was conducted using R Software (R version 3.5.3). Correlation analysis was checked between each predictor variable with response variable and among predictor variables. Generalized Linear Model (GLM) [29] was applied to predict or explain the selection and documentation of the most preferred fuelwood species in the study area.

**Table 1:** Gender characteristics of sampled informants at Boset District

*Kebeles*	Male	Female	Total
Borchota	18	14	32
Geri	21	11	32
Tedecha	20	12	32
Total	59	37	96

## Results and discussion

This particular study was focused on providing scientific evidence for ethnobotanical assessment, identification and prioritization of high fuelwood producing plant species with short-rotation period at Boset District. This, in turn, evaluates their calorific value and combustion characteristics at laboratory so as to solve the high demand of fuelwood problem and contributes to the sustainable management and use of resources through the application of silviculture.

### Socio-demographic characteristics of informants

During ethnobotanical study, data were collected from three selected potential fuelwood producing *Kebeles* at Boset District namely Borchota, Geri and Tedecha that consisting of 7 *Kebele* villages (*Gots*). These were Addis Ketema, Hadhecha, Hadhecha Huletegna, Harabona, Sogido, Bekebishan and Tedecha Huletegna. A total of 96 informants comprising 59 men and 37 women between the ages of 18 and 81 were sampled and participated (Table 1). Most of the informants (99%) were categorized under the medium wealth class, while the remaining were grouped under low income informants.

Out of sampled informants, the youth and adults comprised 90.6% (Table 2). Out of this, 86.4% contributed by men, while 97.3% shared by women. This indicated that most of the informants were under productive age and hence actively involved in firewood collection and/or charcoal making as well as management and utilization practices. Selected informants in the studied District indicated that 67.7% of the informants were engaged on this activity for more than 10 years, while 19.8% of them involved up to 10 years. At the same time, above 78% of the informants were married, while the remaining were single. The average family size of the sampled respondents was 4.63 children, with minimum and maximum family size of 0 and 12, respectively. Of these, over 43% of the informants were illiterate in their educational background, while 19.8% of them attended elementary school (Grade 1–6). This was followed by 17.7% of them, who attended secondary school (Grade 7–12) education or who completed adult education, each. On the other hand, 72.9% of the informants were lived for more than 20 years in the areas and most of them were familiar to forest and forest resources. Overall, the study result indicated that the socio-demographic factors were considerably contributed for firewood collection and/or charcoal making at Boset District.

**Table 2:** Age category of the informants at Boset District

Age category	Age range	Men	Women	Total
Youth	18–29	19	9	28
Adult	30–60	32	27	59
Elder	61–99	8	1	9
Total	18–99	59	37	96

### Socio-economic characteristics of the informants

Ethnobotanical study conducted in three *Kebeles* at Boset District revealed that informants diversify their income from different sources besides to provide their household consumption. These mainly included income from agricultural products; animal husbandry as well as income from firewood collection and charcoal production (Table 3). The study result indicated that among the studied *Kebeles*, Borchota *Kebele* had the highest annual cash income (35.89%) from different sources, followed by Geri *Kebele,* which accounted for 33.08%. The annual average informants’ cash income per household and its percentage contribution earned from different sectors is listed down in Table 3 as follows.

**Table 3:** Average annual informants’ cash income source per household from different sectors at Boset District

*Kebeles*	Agricultural crops (Birr)	Livestock husbandry (Birr)	Firewood collection (Birr)	Charcoal production (Birr)	Total (Birr)	%Age	Rank
Borchota	380.73	142.71	247.40	385.73	1156.57	35.89	1
%Age	32.92	12.34	21.39	33.35			
Rank	2	4	3	1			
Geri	450	196.88	159.38	259.90	1066.16	33.08	2
%Age	42.21	18.47	14.95	24.38			
Rank	1	3	4	2			
Tedecha	275	243.75	189.58	291.67	1000	31.03	3
%Age	27.50	24.8	18.96	29.17			
Rank	2	3	4	1	3222.73		

The exchange rate during data collection was 1 Ethiopian Birr (Birr) = $0.0436 USD

#### Cash income from agricultural products and livestock raising

Data analysis showed that nearly 99% of the informants were farmers and they generate income from different agricultural crops. In the same way, 12.5% of the informants also engaged on livestock husbandry side by side with farming activity. Therefore, most of the informants generated money by selling their agricultural products and livestock in addition to their household subsistence. They earned at least an average annual income of 275 and 142.71 Birr per household from agricultural crops and livestock sale, respectively. These agricultural products were agricultural crops (e.g. maize, *teff (Eragrostis tef)*, sorghum, beans, barley, etc.) and vegetables and fruits (e.g. potato, sugar beet, carrot, onion, etc.). Source of income from livestock sale also included fattened ox, sheep, goat, cow, hen, horse, donkey and mule. Of the total informants, majority of them (64.6%) owned 1–13 ha of land for cultivation of different agricultural crops and raising livestock. In contrast, the remaining informants possessed less than 1 ha of land, while others didn’t have their own land and cultivate crops by renting the land (contract). However, the area was frequently affected by recurrent drought and characterized by erratic rainfall. For instance, some of the informants 7.3% showed that for the last three or four years cultivated crops were failed due to frequent drought occurrence and shortage of rain. As a result, they often faced a problem to subsidize their household income and livelihoods with Government Funded and other supporting aid. Likewise, a similar trend was investigated in the study area by Moroda et al. [30], who found that 26.5% of respondents were mostly food insecured and thereby they subsidize their food by borrowing (9.7%) or through aid (17.9%). Consequently, they look for other alternative income generating activities including firewood collection and/or charcoal production from the surrounding woodland vegetation. Due to this reason, source of income generated from cultivation of agricultural crops was the second in Borchota and Tedecha *Kebeles*. Income source from livestock raising also contributed almost the least across all *Kebeles*.

#### Income diversification from firewood collection and/or charcoal production

Analysis of field data indicated that informants in the study area engaged on various income generating activities hand in hand with farming activities. These were mainly on forest and forest products such as timber harvesting, firewood collection and/or charcoal making, beehive hanging and honey collection, harvesting medicinal plants for traditional remedies as well as collecting gum and resin products. However, source of income generated from charcoal making ranked first both in Borchota *Kebele* (33.35%) and Tedecha *Kebele* (29.17%). A similar study conducted by Vollmer et al. [31] indicated that 70% of the cash income earned by households is from charcoal production in southern Mozambique. In relation to this, almost 50% of the world’s population as well as about 81% of households in the region of Sub-Saharan Africa depend on wood energy, *i.e*., fuelwood and charcoal for cooking purposes [32]. On the other hand, collection of firewood was the list income generating activity in Tedecha *Kebele* (29.17%), followed by Borchota *Kebele* (21.39%). Similarly, the same trend was observed for informants’ preference to charcoal production (2^nd^ ranked) rather than firewood collection (least ranked) in Geri *Kebele*. This finding most probably suggests that informants in the studied *Kebele*s were mainly depending on charcoal production instead of firewood collection since they generate better income source. Pervious study further assured that there is a shift from fuelwood collection to charcoal production for cooking and heating purposes due to fast growth of urbanization [33]. Furthermore, 97.9% of the informants in the studied *Kebele*s engaged on firewood collection and/or charcoal making. This figure showed that informants were directly relied on firewood and/or charcoal for cooking, heating and lighting purposes using traditional open fire or low efficiency traditional cooking stoves. This finding is in agreement with other previous study by Feyisa et al. [34], who reported that all of the sampled informants at Gechi District rely on fuelwood for cooking and lighting. Consequently, these authors further reported that 6529.9 tons/ha fuelwood on average is consumed by the community in the study area. Likewise, electric access in the rural setting is around 5% so that fuelwood has a significant contribution to rural population [2]. However, 54.2% of them used other sources of energy such as electric, fossil fuel, solar, crop and animal residues as main or alternative energy sources. In the same way, alternative energy sources including cow dung, crop residues, kerosene and electricity are served in towns and rural areas at Ziway [35]. As a result, income generated from these sources had significantly contributed to rural households to subsidize their livelihoods. Table 4 showed that 88.5% of the informants at Boset District involved in firewood collection, while 90% of them practiced charcoal making. Of this, more than half of the informants (55.2%) were men, who engaged by providing firewood and charcoal to the market. However, most of the time women and children spend most of their time to collect firewood from long distances and less accessible areas. But in this particular study higher contribution of men than women probably suggests the task requires more labour intensive under harsh environmental conditions. On the other hand, men participated in charcoal making since it requires a lot of traditional processes and labor intensive as well. Among the sampled *Kebeles*, more firewood collection and charcoal making was contributed in Geri and Tedecha *Kebeles*.

**Table 4:** Number of informants involved in firewood collection and charcoal production at Boset District

*Kebeles*	Firewood collection		Total	Charcoal production		Total
	Men	Women		Men	Women	
Borchota	16	11	27	15	11	26
Geri	19	10	29	19	11	30
Tedecha	18	11	29	19	11	30
Total	53	32	85	53	33	86

The average annual income from firewood collection and/or charcoal making, in turn, indicated that a total of 1,533.60 Ethiopian Birr per household was earned by providing to market areas including around their village, along the Addis Ababa asphalt highway or Olenchit town (Table 5). Of these, 36.7% of firewood collection and 46.9% of charcoal making was contributed by women households. In line with this, the highest collection of firewood (41.5%) as well as charcoal production (41.2%) was supplied to the market to satisfy fuelwood demand by Borchota *Kebele*. On the contrary, the least annual income from both sources (26.7% of firewood collection and 27.7% of charcoal production) was generated by Geri *Kebele*.

**Table 5:** Average annual informants’ income in Ethiopian Birr per household from firewood collection and charcoal production at Boset District

*Kebeles*	Firewood collection		Total	Charcoal production		Total
	Male	Female		Male	Female	
Borchota	149	98.4	247.4	140.9	244.8	385.7
Geri	115.1	44.3	159.4	169.3	90.6	259.9
Tedecha	113.5	76	189.5	187.5	104.2	291.7
Total	377.6	218.7	596.3	497.7	439.6	937.3

### Informants’ attitude and perception towards firewood collection and charcoal production at Boset District

The study result showed that local communities depend on various forest and forest products from the surrounding woodland vegetation. These were firewood collection and charcoal making; timber harvesting; beehive hanging and honey collection; traditional medicinal plants harvesting; harvesting of bamboo, wild fruits as well as gum and resin products. Among these, almost all of the informants (97.9%) engaged on firewood collection and/or charcoal making as compared to other forest and forest products. Findings from the study area corroborate this investigation and showed that charcoal sale is the main source of income for 83% of respondents [36]. With this, majority of the informants (72.9%) engaged on this activity for both household subsistence and to diversify their source of income. In contrast, 23.9% of them were required it only for household subsistence. Furthermore, collection of firewood contributed 55.3% of the average annual source of income at Boset District (Table 4). Similarly, an earlier study conducted in and around the semi-arid Awash National Park by Bahru et al. [23] revealed that firewood was the major source of energy, which accounted for 73% and an income generating activity in the livelihoods of many rural dwellers. In turn, about 30% of charcoal making is accountable for the forest degradation [36]. Hence, majority of local communities use fuelwood to cook their food, heat and light up their houses [23]. At the same time, contribution of charcoal production (86.9%) (Table 3) attributed to most probably due to higher potential of *Acacia* species in the studied *Kebeles*. A study conducted by Balemie et al. [37] in Fentalle area, in turn, found out that firewood collection and charcoal making contributed 17.9 and 18.5%, respectively. According to Zerihun and Mesfin [38], the Rift Valley vegetation is an important source of charcoal making for the nearby towns and Addis Ababa. As a whole, a study from 2015 reported that wood is harvested for the supply of 115 million m^3^ firewood and 5.4 million m^3^ charcoal [2]. In addition, traditional and low efficiency cooking stoves and open cooking fire in the rural areas further contributes for the high demand of fuelwood species and thereby depletion of forest resources. This is because open cooking fire and poorly designed traditional cooking stoves may have 3–5% lower energy efficiency than high efficiency improved cooking stoves [9]. Therefore, a high efficiency improved cooking stoves instead of traditional low efficiency stoves and open cooking fire can save the loss of energy from fuelwood [9].

With this understanding, most of the documented fuelwood producing plant species were indigenous or native (76%) to the area, while the remaining species were introduced or exotic to the study area. More than 33% of the informants described that they used introduced species for fuelwood consumption, while 7.3% of them did not use it. Analyzed data further showed that 27% of the informants pointed out that they used these species so as to control their invasiveness and hence their spread to the area. Again, their easier availability, their importance for source of fuelwood species and shortage of preferred fuelwood species in the study area also made informants to use introduced species. According to various sources and informants’ suggestion, these exotic species were introduced to the study area due to various reasons at various time. For instance, *Prosopis juliflora* introduced to arid and semi-arid areas to rehabilitate the dryland areas. *Parthenium hysterophorus* also introduced through food aid with agricultural crops. Other introduced species, which are used for fuelwood species also comprised *Azadirachta indica*, *Calotropis procera, Cryptostegia grandiflora, Lantana camara* and *Senna occidentalis.* Out of these, *P. juliflora* is served as a good quality charcoal producing species. A study also reported by Onekon and Kipchirchir [39] showed that *P. juliflora* is most preferred by 10% of the respondents in Kenya. This is because the species is profitable and not a banned tree species for charcoal production. In the same way, *A. indica* and *C. grandiflora* often used as charcoal producing species, while the rest introduced specis were collected and used as firewood especially during dry season, when fuelwood was scarce. On the other hand, above 6% of the informants were further relied on other sources such as timber harvesting; honey collection; traditional medicinal plants harvesting as well as collecting gum and resin. During data collection, best quality parameters commonly practiced by the local communities to identify and prioritize species for firewood collection and/or charcoal making were listed. These included superior combustion characteristics (giving good energy, heat and light); higher amount of energy, heat and light produced per dry wood; lower pollution problems (little or no smoke or soot and ash contents); formation of little/no spark during heating and lighting; easier to cut and split the wood; production of high energy with longer time as well as heavy wood's dry weight per volume (density) with minimum moisture content; fuelwood species are easily accessible to the area and hence easier to collect firewood and make charcoal. This finding was corroborated with earlier study conducted by Bahru et al. [23], who indicated that selection of firewood mainly relies on availability, burning quality, little/no smoke/soot production and moisture content. Some of these fuelwood characteristics also reported by FAO [9, 10] and ILO [11]. Overall, a total of 25 fuelwood producing plant species were identified and listed at Boset District during this field study (Appendix 1).

### Management and sustainable utilization of fuelwood species at Boset District

Informants’ interview clearly showed that majority of them (83%) indicated that local communities widely collect firewood and produce charcoal from natural forests around the study area. Consequently, all informants (96 of them) agreed that the most preferred and selected fuelwood species in the study area were declining in terms of their distribution and population size from time to time due to various anthropogenic factors. Of these, 78% of the informants mentioned two reasons as the main cause for species decline at Boset District. These were first local communities use these species as the only energy source and the demand for fuelwood use becomes increased. Second, local communities commonly practiced the traditional open fire or poorly designed traditional cooking stoves for cooking, heating and lighting purposes. This is attributed to the loss of most of the fuelwood species due to the high fuelwood demand in the area. On the other hand, 26% of the informants stated that the population size of fuelwood species was declined over time at alarming rate. This is because in the study area overexploitation of fuelwood species particularly *Acacia* species for charcoal production is commonly practiced. For instance, a study conducted by Bahru et al. [23] around the study area, showed that during both preference ranking and pairwise comparison for charcoal production, all species are classified under *Acacia* species. By contrast, according to Feyisa et al. [34] report at Gechi District, the most preferred and harvested fuelwood species are *Syzygium guineense, Maesa lanceolata* and *Albizia gummifera.* This might be associated with the limited availability of *Acacia* species in this particular area due to forest depletion over long period of time. Others also listed that due to the high demand for fuelwood, the supply was increased to satisfy the local communities need. However, other study showed that overgrazing/over browsing and deforestation for various uses are the major threats to fuelwood species [23].

Nevertheless, 29.2% of the informants explained that local communities manage and conserve the most preferred and selected fuelwood species in the study area using various ways. Some of these were plant seedlings; protect species from cutting; replant the species around homesteads, farmlands and farm boundaries once cut/harvested for the required use; proper collection of the product as well as demarcating and protect/conserve the forest. For instance, some of the local communities satisfy their demand for fuelwood products through planting *Acacia tortilis, Eucalyptus species, Ziziphus mucronata, A. robusta* and *A. indica* plant species, which is similarly reported for the conservation and management of some of the species by Bahru et al. [23], Balemie et al. [37] and Hunde et al. [40]. According to some informants this species can be harvested within 3–7 years after planted for fuelwood purpose. Collected data as well as researcher observation during data collection indicated that local communities planted these species around homesteads and fences, farmlands and farm boundaries as well as other marginal lands. They selected these areas mainly due to the fact that it enhances soil fertility, soil and water conservation as well as easy for proper management of seedlings. Others also preferred to grow plants around homesteads and fences, farm boundaries and other marginal areas due to shortage of farmland or such tradition was commonly practiced by the local communities. In general, this trend revealed that local communities at Boset District have the traditional system to manage and conserve the natural resources, as also similarly reported by Hunde et al. [40]. Therefore, future plantation establishment and management through the application of suitable silvicultural practices (e.g. spacing, thinning, pruning and coppice management) will be helpful to address the high demand of short-rotation fuelwood species within three or four years.

### Identification, prioritization and selection of the most preferred fuelwood species at Boset District

During ethnobotanical study, simple preference ranking followed by species pairwise comparison indicated that the best three most popular or commonly preferred fuelwood producing species were selected. Accordingly, *A. senegal* was the most commonly preferred high fuelwood species, which ranked 1^st^ with a total of 267 scores given by key informants (Table 6). This was followed by *A. tortilis* and *A. robusta*, which was selected with 244 and 42 total scores, respectively.

**Table 6:** Identification, prioritization and selection of the most popular fuelwood species at Boset District

Scientific name	Family name	Habit	Local name	Rank after pairwise comparison
*A. senegal* (L.) Willd	*Fabaceae*	Shrub	Sephensa/Kertefa (Or); Kontir (Am)	1
*A. tortilis* (Forssk.) Hayne	*Fabaceae*	Tree	Tedecha (Or)	2
*A. robusta* Burch	*Fabaceae*	Tree	Wanigayo (Or)	3

Am, Amharic language; Or, Afan Oromo language

### Major opportunities and challenges for the plantation development, proper management and sustainable utilisation of fuelwood species

In Boset District, there are many promising opportunities for small-scale plantation development, management and sustainable utilisation on most preferred and selected fuelwood species. Some of these opportunities listed during informants’ interview were provision of alternative energy and income source; untapped fuelwood resource; fast growing and short-rotation fuelwood species as well as high demand and fair price of products. Of these, about 99% of the informants listed the first three opportunities for small-scale plantation development, proper management and sustainable utilisation on most preferred and selected fuelwood species. Despite this fact there are major challenges and constraints for plantation development, management and sustainable utilisation on the most preferred and selected fuelwood species in the study area. These major challenges were listed as follows: limited awareness on management and conservation of the resources (training, education, experts support and supervision, etc.); shortage of land to expand the resource; lack of market access and value chains for the product. Others included limited awareness on the use of the resource; shortage of quality and amount of seeds and seedlings; shortage of labor for quality and quantity product; lack of facilities and logistics to produce the product; government limited focus on the resource; high cost of labour and budget for resource management and conservation as well as various anthropogenic (agricultural and investment expansion, charcoal production, overgrazing/browsing, forest fire) and natural factors (drought). Some of the aforementioned threats to fuelwood species further reported by Bahru et al. [23] and Balemie et al. [37], in the semi-arid parts of the Rift Valley area. Of these challenges mentioned above, the first three were chosen by 96.9, 54.2 and 50% of the informants, respectively (Table 7).

**Table 7:** Major challenges for the proper management, sustainable utilisation and plantation development of fuelwood species at Boset District

Nos	List of major challenges	% of informants listed the challenge	No	List of major challenges	% of informants listed the challenge
1	Limited awareness on management and conservation of the resources	96.9	6	Shortage of quality and amount of seeds and seedlings	31.3
2	Various anthropogenic (agricultural and investment expansion, forest fire) and natural factors	54.2	7	Government limited focus on the resource	27.1
3	Limited awareness on the use of the resource	50	8	High cost of labour and budget for resource management and conservation	24
4	Shortage of labor for quality and quantity product and lack of facilities and logistics to produce the product	37.5	9	Lack of market access and value chains for the product	24
5	Shortage of land to expand the resource	34.4			

### Model development on selection of high fuelwood producing species at Boset District

Major predictor variables were identified and a GLM was developed to predict the correlation of various socio-economic and demographic features of informants’ with the selection of high fuelwood producing plant species at Boset District. A response variable and 13 major predictor variables were listed in Table 8 and described in detail as follows.

**Table 8:** Summary of the developed GLM for response and predictor variables on high fuelwood producing plant species at Boset District

List of variables	Variable type	Correlation coefficient (r)	Standard error	z-value	Pr( >|z|)
Selection of high fuelwood producing plant species at Boset District (intercept)	Response	3.08e^+00^	3.49e^−01^	8.84	< 2e^−16^***
Sampled *Kebeles* at Boset District	Predictor	1.57e^−01^	2.43e^−02^	6.45	1.1e^−10^***
Informant’s gender		− 5.74e^−02^	3.72e^−02^	− 1.54	0.12
Informant’s age		2.85e^−03^	1.46e^−03^	1.96	0.05*
Informant’s wealth class		1.94e^−01^	1.58e^−01^	1.23	0.22
Informant’s marriage status		− 1.83e^−02^	5.82e^−02^	− 0.32	0.75
Informant’s educational background		− 7.03e^−03^	9.80e^−03^	− 0.72	0.47
Informants’ main source of livelihoods		− 3.38e^−02^	1.23e^−02^	− 2.74	6.13e^−03^**
Informants’ years of experience in firewood collection &/or charcoal making		− 2.54e^−02^	1.30e^−02^	− 1.96	0.0498*
Informants’ source of energy for cooking, heating and lighting purposes		1.17e^−02^	4.99e^−03^	2.34	0.02*
Informants income source generated from firewood collection &/or charcoal making		4.78e^−05^	1.24e^−05^	3.85	1.21e^−04^***

The statistical significance difference between predictor and response variables is illustrated as follows: “***” 0.001, “**” 0.01, “*” 0.05, “.” 0.1, and “ ” 1

**Fig. 2:**
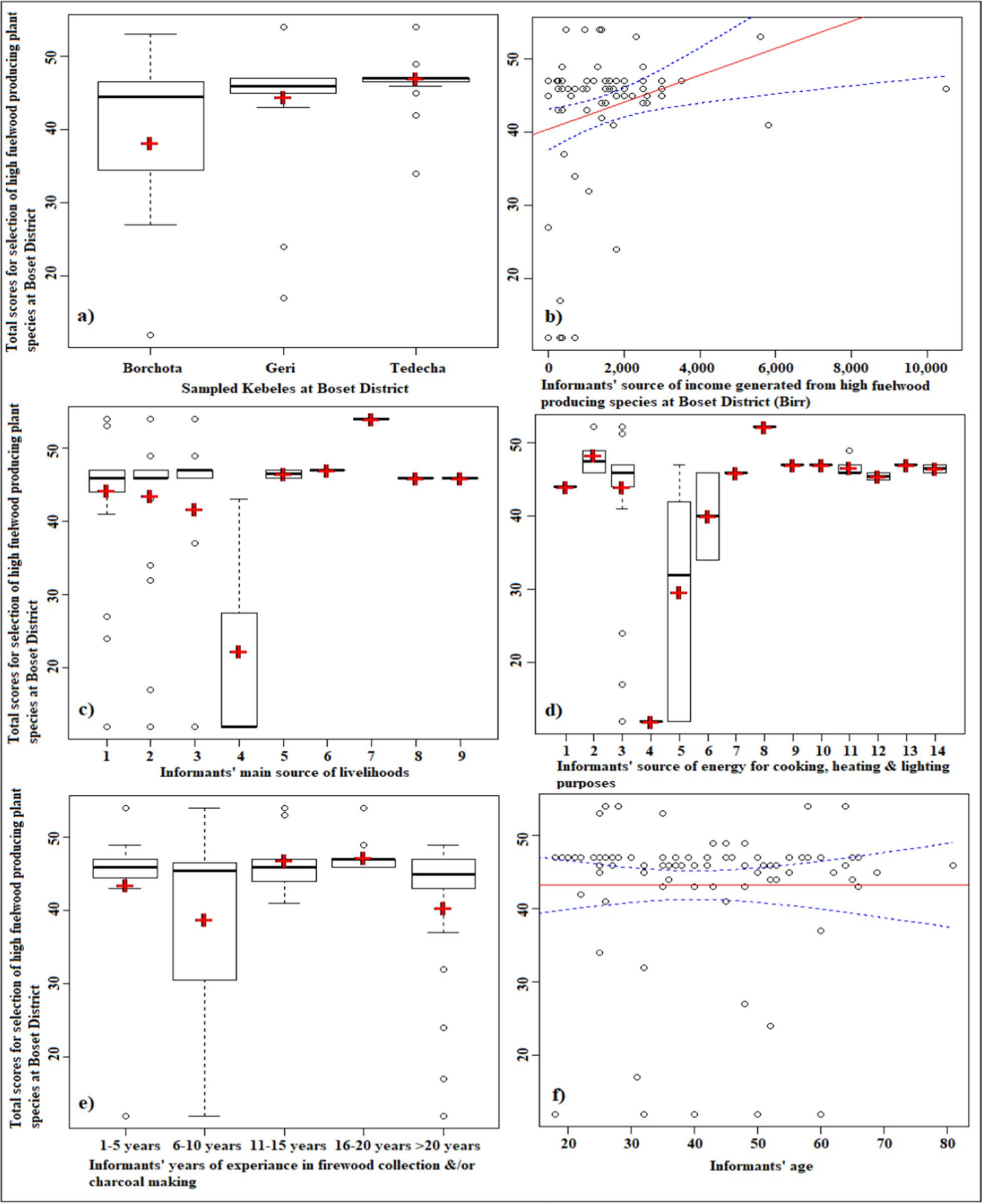
Predicted GLM on selection of high fuelwood producing species at Boset District: **a** among sampled *Kebeles*; **b** informants’ source of income generated from firewood collection and/or charcoal making; **c** informants’ main livelihoods; d) informants’ source of energy for cooking, heating and lighting purposes; **e** informants’ number of years of experience in firewood collection &/or charcoal making; and **f** informants age. Solid regression line in **b**, **f** illustrates model predictions for sampled *Kebeles* and informants’ age and the broken lines indicate 95% confidence interval. The horizontal line inside each Boxplot (**a**, **c**–**e**) shows the median; crossbar indicates the mean; broken lines outside each boxplot demonstrate the whiskers and the dots are the outliers. Numbers (1–9) in **c** shows: (1) farmers; (2) farmers, firewood collector and charcoal producer; (3) farmers and firewood collector; (4) farmers and pastoralists; (5) farmers, gum and resin collector; (6) farmers, pastoralist and firewood collector; (7) pastoralist, firewood collector and charcoal maker; (8) farmers, pastoralists, employer, firewood collector and charcoal producer; and (9) farmers, pastoralist, employer and firewood collector. Numbers (1–14) in **d** indicates: (1) electric; (2) electric and firewood; (3). firewood; (4). charcoal and firewood; (5) firewood and animal manure; (6) electric, charcoal and firewood; (7) animal manure; (8) electric, firewood and animal manure; (9) electric, charcoal, firewood and animal manure; (10) electric, charcoal, firewood, crop residue and animal manure; (11) charcoal, firewood, kerosene, crop residue and animal manure; (12) charcoal, firewood, solar energy, crop residue and animal manure; (13) electric, charcoal, firewood, kerosene, crop residue and animal manure; and (14) charcoal, firewood, kerosene, solar energy, crop residue and animal manure

The developed GLM demonstrated that sampled *Kebeles* and informant’s source of income generated from firewood collection and/or charcoal making was positively and significantly (*p* < 0.001) associated to identification, prioritization and selection of high fuelwood producing plant species (Table 8; Fig. 2a, b). In the same way, informants’ main source of livelihoods also significantly (*p* < 0.01) correlated but negatively (Table 8; Fig. 2c). On the other hand, source of energy, informants’ age and experience were significantly related (*p* < 0.05) although years of experience was negatively correlated (Table 8; Fig. 2d–f).

## Conclusions and recommendations

Collected data indicated that 97.9% engaged on firewood collection and/or charcoal making as compared to other forest and forest products. Out of these, 88.5% of the informants involved in firewood collection, while 90% of them practiced charcoal making. The average annual income from firewood collection and/or charcoal making, in turn, indicated that a total of 1,533.60 Ethiopian Birr per household was earned. With this, *Acacia senegal, Acacia tortilis* and *Acacia robusta* were the three best prioritized and selected fuelwood species for high quality fuelwood provision at Boset District. The GLM further confirmed that sampled *Kebeles* and informant’s source of income significantly related to the selection of species at *p* < 0.001, while informants’ main source of livelihoods correlated at *p* < 0.01. Higher efficiency to provide energy, heat and light; little or no smoke or soot, spark and ash contents; easier to cut and split the wood, heavy wood's dry weight per volume (density) with minimum moisture content as well as easier availability were some of the main selection criteria for firewood and/or charcoal species in the study area. At the same time, there is also a growing practice in the community to use improved cooking stoves for cooking, heating and lighting to save the energy. Local communities should also use improved cooking stoves such as *Mirt* and *Gonzie* for baking *Injera* while *Tikikil* and *Lakech* for other services instead of traditional open fire cooking materials to save energy, firewood supply and/or charcoal production and even to avoid accidental fire incidence. Furthermore, to save the loss of energy during carbonisation process, charcoal makers should practice improved earth mound kiln instead of traditional earth mound kiln. Future plantation establishment and management through the application of suitable silvicultural practices (e.g. spacing, thinning, pruning and coppice management) will be helpful to address the high demand of short-rotation fuelwood species within three or four years. The most preferred fuelwood species (*A. senegal, A. tortilis* and *A. robusta*) in the area should be further tested at laboratory to evaluate the energy value for further promotion and developing management strategy.

## Data Availability

Data in the current study are available from the corresponding author upon reasonable request from Central Ethiopia Environment and Forest Research Center (CEE-FRC)/Ethiopian Environment and Forest Research Institute (EEFRI).

## Appendix 1: List of fuelwood species at Boset District, central Ethiopia

**Table:**

Nos.	Scientific name	Family name	Habit	Status	Local name
1	*Acacia albida* Del	*Fabaceae*	Tree	Indigenous	Gerbi (Or)
2	*Acacia dolichocephala* Harms	*Fabaceae*	Tree	Indigenous	Kesele (Or)
4	*Acacia oerfota* (Forssk.) Schweinf	*Fabaceae*	Shrub	Indigenous	Ajo (Or)
5	*Acacia robusta* Burch	*Fabaceae*	Tree	Indigenous	Wanigayo (Or)
6	*Acacia senegal* (L.) Willd	*Fabaceae*	Shrub	Indigenous	Sephensa/Kertefa (Or); Kontir (Am)
7	*Acacia tortilis* (Forssk.) Hayne	*Fabaceae*	Tree	Indigenous	Tedecha (Or)
8	*Azadirachta indica* A. Juss	*Meliaceae*	Tree	Introduced	Genete/Miliya (Or)
9	*Balanites aegyptiaca* (L.) Del	*Balanitaceae*	Tree	Indigenous	Bedeno (Or/Am); Jemo (Am)
10	*Berchemia discolor* (Klotzsch) Hemsl	*Rhamnaceae*	Tree	Indigenous	Jejeba (Or/Am)
11	*Dichrostachys cinerea* (L.) Wight & Arn	*Fabaceae*	Shrub	Indigenous	Ader/Hate (Or)
12	*Calotropis procera* (Ait.) Ait.f	*Asclepiadaceae*	Shrub	Introduced	Gura Hare/Bun Gedhe (Or); Tobya/Kinbo (Am)
13	*Combretum hartimanium* R. Br. ex G. Don	*Combretaceae*	Tree	Indigenous	Debelocha (Or)/Tilem (Am)
14	*Commiphora habessinica* (Berg) Engl	*Burseraceae*	Shrub	Indigenous	Hamecha (Or); Anqwa (Am)
15	*Cryptostegia grandiflora* Roxb. ex R. Br	*Asclepiadaceae*	Shrub	Introduced	Fenkol/Hakonkol (Or)
16	*Euclea racemosa* Murr. subsp. *schimperi* (A. DC.) White	*Ebenaceae*	Shrub	Indigenous	Miecha (Or); Dedeho (Am)
17	*Grewia bicolor* Juss	*Tiliaceae*	Shrub	Indigenous	Harorecha (Or); Teye (Am)
18	*Lantana camara* L	*Verbenaceae*	Shrub	Introduced	Afan Dubra/Midhan Dubra (Or); Yewof Kolo (Am)
19	*Olea europaea* L. subsp. *cuspidata* (Wall.ex G. Don) Cif	*Oleaceae*	Tree	Indigenous	Ejersa (Or); Weyra (Am)
20	*Parthenium hysterophorus* L	*Asteraceae*	Herb	Introduced	Feremsisa/Ferme/Fermi Basa/Biye Base/Dhukub Lefa (Or)
21	*Premna resinosa* (Hochst.) Schauer	*Lamiaceae*	Shrub	Indigenous	Argecha (Or)
22	*Prosopis juliflora* (Sw.) DC	*Fabaceae*	Shrub	Introduced	Shola/Yeweyane Zaf (Or)
23	*Senna occidentalis* (L.) Link	*Fabaceae*	Herb	Introduced	Shewshewe (Or)
24	*Terminalia brownii* Fresen	*Combretaceae*	Tree	Indigenous	Birecha (Or); Weyba (Am)
25	*Ziziphus mucronata* Willd	*Rhamnaceae*	Tree	Indigenous	Kurkura (Or/Am)
